# Temperature and Strain Rate Effects on the Uniaxial Tensile Behaviour of ETFE Foils

**DOI:** 10.3390/polym14153156

**Published:** 2022-08-02

**Authors:** Felix Surholt, Jörg Uhlemann, Natalie Stranghöner

**Affiliations:** Institute for Metal and Lightweight Structures, University of Duisburg-Essen, 45141 Essen, Germany; joerg.uhlemann@uni-due.de (J.U.); natalie.stranghoener@uni-due.de (N.S.)

**Keywords:** ETFE foils, base material, temperature, strain rate, uniaxial tensile tests, model parameter, nonlinear material model

## Abstract

With the first use of ETFE foils in building structures in the 1980s at the Burgers’ Zoo in Arnhem, Netherlands, the implementation of ETFE foils in roof and façade systems in large-span structures has become steadily more prominent. To safely design ETFE foil structures, their mechanical behaviour has to be fundamentally understood. Until now, several research studies have been published investigating this material’s behaviour. However, the parameters influencing these plastic’s mechanical behaviour, such as the strain rate or the test temperature, have only been investigated separately but not simultaneously. In this contribution, an analytical model is presented which describes the mechanical behaviour of ETFE foils under varying test temperatures and strain rates simultaneously. The material model has been checked against experimental results achieved for materials from three different international producers and two different commonly used foil thicknesses with significant differences in their mechanical responses (so that it can be assumed that the international market is represented). In the first step, uniaxial tensile tests on strip specimens were performed to describe the nonlinear and viscoelastic temperature- and strain rate-dependent material behaviour under uniaxial tension. The achieved stress-strain curves exhibited, as expected, the two commonly so-called yield points, which can be taken as separators for three different material stages: viscoelastic, viscoelastic-plastic, and viscoplastic. In the second step, by separating the uniaxial tensile response into these three stages, two interdependent functions could be derived based on the well-known Ramberg-Osgood material model to simulate the viscoelastic and viscoelastic-plastic material behaviour of ETFE foils. For this purpose, analytical functions were developed to calculate the model parameters considering the influence of the test temperature and the test speed. It can be shown that the newly developed analytical material model fits well with the experimental results. With the use of the derived nonlinear material model, design engineers can predict the material’s mechanical behaviour considering the environmental conditions on site while maintaining independence from the material’s supplier.

## 1. Introduction

### 1.1. General

In the field of membrane structures, architectural and structural engineering requirements are closely linked. In the 1950s, technical textiles were used for the first time as wide-span surface structures. A comparatively new type of membrane structures, foil structures, emerged in the 1980s. Both technical textiles and foils have a very low weight per unit area, which is why they are used for wide and support-free spans of surfaces. In most cases, the tensile stressed membranes are used for representative buildings. For example, stadiums are predestined structures on which wide-span foils or fabrics can be mounted; both are used within the roof structure and/or in the façade.

For foil structures, foils made of fluorine-based plastic ethylene tetrafluoroethylene (ETFE) are commonly used. They excel due to their light weight (1.75 g/cm^3^) and their transparency. Regarding transparency, ETFE foils excel at transmitting roughly 95% of light, while the transmission quotient of glass lies between 83% and 90% depending on the plate thickness [1,2,3]. With the given properties, ETFE foils are predestined for representative buildings.

Regarding the load-bearing behaviour of membrane structures, textile fabrics and foils can only bear tensile forces and no bending or compressive forces. Therefore, membrane structures require prestresses to enable their load-bearing capacities. In general, two different kinds of structures can be distinguished: (a) synclastic, double-layer or multi-layer cushion structures, which are typically prestressed with an internal air pressure forming enclosed envelopes; and (b) anticlastic or flat, mechanically prestressed single-layer structures. In the ultimate limit state and serviceability limit state design of those structures, the different shapes and kinds of prestress applications have to be considered. In cushions, the application of the prestress using internal air pressure allows the compensation of plastic deformations up to a certain extent. The required pretension of the foils is achieved by the internal pressure independently from the air volume. Usually, an internal pressure of 250 Pa can be used for pretensioning the cushion foils. For mechanically prestressed single-layer structures a typical characteristic is that they cannot compensate any plastic deformations arising from external loads. Plastic strains lead to a decrease of the applied prestress so that, in the worst case, the structural integrity cannot be ensured anymore (which might lead to a structural collapse).

In the newly developed prCEN/TS 19102 [4], these two structural types of membrane structures—synclastic and anticlastic/flat—and their prestress application are considered in the design procedures which are expressed within the new so-called k-factor concept used in the ultimate and serviceability limit state design. The k-factor concept is based on the German A-factor concept developed by Minte in the 1980s [5]. Within the k-factor concept, the characteristic ultimate tensile strength f_u_ of ETFE foils is modified depending on the design condition. Herein, different load speeds, load durations, and temperatures are considered. For example, the design condition “snow (≤1000 m altitude)” takes into account the influence of an acting temperature of T = 0 °C and the slowly accumulating snow as a long-term load. The k-factors given in prCEN/TS 19102 are based partly on experiences of the experts involved in the development of the prCEN/TS 19102 and partly on specific investigations into the k-factors carried out by the authors of this contribution who were involved in the in the development of the prCEN/TS 19102 as well. For this purpose, the authors could rely on investigations into the uniaxial tensile behaviour of ETFE foils under different test temperatures and strain rates carried out in the frame of the German DFG research project “Characterisation of the Nonlinear Viscoelastic Material Behaviour of ETFE and ECTFE Foils for the Use in Building Membrane Structures” (STR 482/6-1). Since ETFE is a thermoplastic, its mechanical properties and load-bearing behaviour vastly depend on the test temperature and strain rate (e.g., [6,7,8,9,10,11,12,13,14,15,16,17]). As ETFE foils are typically loaded biaxially, the material behaviour under biaxial loads is of interest as well. Consequently, both the uniaxial and biaxial load bearing behaviour of ETFE foils has to be addressed. Anyway, this contribution focuses on the uniaxial tensile behaviour while future publications will focus on the biaxial behaviour under varying test temperatures and strain rates.

### 1.2. Uniaxial Stress-Strain Behaviour of ETFE Foils

**Figure 1:**
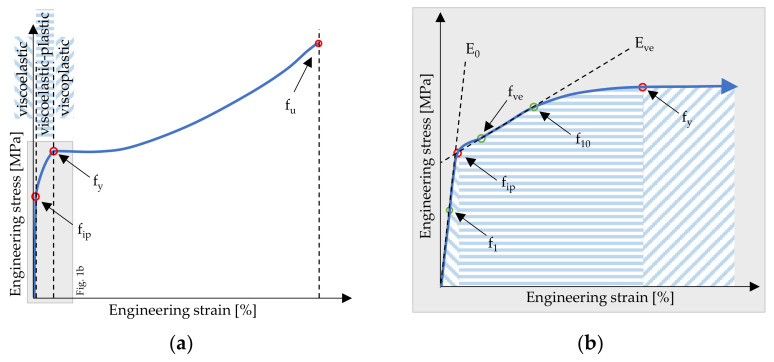
(**a**) Stylized illustration of a uniaxial stress-strain diagram of ETFE foils and (**b**) stylized illustration of a uniaxial stress-strain diagram of ETFE foils at relatively small strains (cut shortly after the yield point f_y_).

The uniaxial tensile behaviour of ETFE foils can be best described by its stress-strain behaviour as presented in a stress-strain diagram in Figure 1a. Herein, two inflexion points are prominent at which the material stiffness drastically changes. These two inflexion points are commonly referred to as the first and second yield point, which separate the mechanical behaviour of ETFE foils under uniaxial tension into three different material stages: viscoelastic, viscoelastic-plastic, and viscoplastic. However, yielding, as defined in EN ISO 527-1 [18], marks the point in a stress-strain diagram at which the engineering strain increases without any further increase of the engineering stress. For this reason, the first inflexion point cannot be called “first yield point” but is called “inflexion point” f_ip_ in this paper. Only the second inflexion point is called the “yield point” f_y_. Herewith, a stress-strain diagram from a uniaxial tensile test of an ETFE foil exhibits three characterising points: (1) the inflexion point f_ip_; (2) the yield point f_y_; and (3) the ultimate tensile strength f_u_ at which the ETFE foil breaks.

However, in everyday engineering applications, mainly the viscoelastic and viscoelastic-plastic parts of ETFE foils are relevant so that additional characteristic material parameters are of interest to typify ETFE foils. These additional characteristic material parameters are the model parameter f_ve_, the stress at 1% strain f_1_ and the stress at 10% strain f_10_ as shown in Figure 1b and marked by green circles. f_ve_ marks the point at which the second linear part in a uniaxial stress-strain diagram starts which is characterised by the stiffness modulus E_ve_. E_ve_ describes the stiffness of the viscoelastic-plastic part of the stress-strain behaviour.

In the past, several investigations into the development of an analytical model for describing the uniaxial stress-strain behaviour of ETFE foils were carried out by, e.g., Hu et al. [8,13], Zhao et al. [9,10], and Beck [17]. However, all of these models have in common, that they do not consider temperature and strain rate effects in uniaxial tensile tests simultaneously.

For this reason, an analytical model has been developed by the authors of this contribution for describing the uniaxial stress-strain behaviour of ETFE foils considering both temperature and strain rate effects which has been based on the modified Ramberg-Osgood material model for stainless steel [19,20,21,22]. This newly developed analytical material model has been verified with experimental results achieved from investigations for various ETFE foils and is presented in the following.

## 2. Experimental Investigations into the Uniaxial Tensile Behaviour of ETFE Foils under Various Test Temperatures and Strain Rates

### 2.1. Test Matrix, Material and Specimen Preparation

To derive the influence of various test temperatures and strain rates, uniaxial tensile tests were performed considering four temperatures and five load speeds. The test temperatures were chosen to T_0_ = 0 °C, T_23_ = 23 °C, T_40_ = 40 °C and T_50_ = 50 °C (±2 K for each temperature). The test speeds were chosen to ${\dot{v}}_{10}$ = 10 mm/min, ${\dot{v}}_{50}$ = 50 mm/min, ${\dot{v}}_{100}$ = 100 mm/min, ${\dot{v}}_{200}$ = 200 mm/min and ${\dot{v}}_{500}$ = 500 mm/min, representing different strain rates. Additionally, ETFE foil materials from three different producers and two different foil thicknesses were analysed. Table 1 lists the exemplary physical and chemical properties of ETFE copolymer.

**Table 1:** Exemplary physical and chemical properties of ETFE copolymer [23].

Property	Unit	Value
Density	g/cm^3^	1.67–1.75
Melting temperature	°C	265–270
Coefficient of linear expansion		
Longitudinal (23–55 °C)	10^−5^/K	7–10
Transversal (23–55 °C)	10^−5^/K	-
Water absorption (23 °C)	%	<0.05
Humidity absorption (23 °C, 50% RH)	%	<0.05

Table 2 presents the test matrix. The investigated 100 µm and 250 µm foil materials were provided by AGC Chemicals Europe Ltd., Amsterdam, The Netherlands (Fluon ETFE Film 250NJ-1550NT, Fluon ETFE Film 100NJ-1550NT), Nowofol GmbH & Co. KG, Siegsdorf, Germany (Nowoflon ET 6235 Z in 250 µm and 100 µm) and Textiles Coated Europe GmbH (TCI), Kaarst, Germany. (ETFE AG 250 µm, ETFE AG 100 µm). The materials provided by AGC were shipped from Japan, the materials from Nowofol came from Germany, and the materials from TCI were shipped from USA. Please note, that this order does not reflect the allocation of producers I, II, and III, respectively.

**Figure 2:**
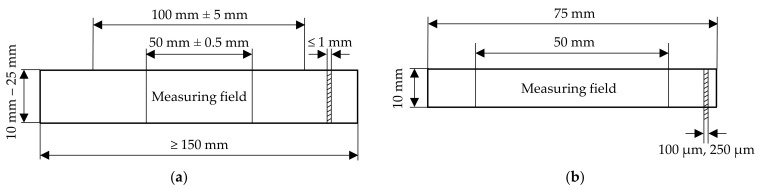
(**a**) Dimensions of a rectangular strip specimen according to [24] and (**b**) dimensions of the used strip specimen.

The uniaxial tensile tests were performed according to EN ISO 527-1 [18] and -3 [24]. EN ISO 527-3 suggests two specimen geometries to derive the mechanical behaviour of foils, rectangular strip specimens and dumbbell specimens. In this study, rectangular strip specimens were used, which are suggested in [24] due to their easy processing. Figure 2a illustrates the dimensions of a rectangular strip specimen specified in [24]. However, due to large breaking strains—which apply for ETFE foils—and therefore the possibility of insufficient length of the traverse path of the testing machine, [24] allows for reducing the initial clamping length of 100 mm to 50 mm. The final specimen dimensions used in the presented study for the uniaxial tensile tests are shown in Figure 2b. Additional and special provisions mentioned in [4] are complied with, regarding a specimen length to width ratio of 5:1. With an initial clamping length of 50 mm, the specimen width was defined to 10 mm. To ensure a proper clamping, specimens with 12.5 mm clamping length on each side were prepared so that the whole specimen length was 75 mm. To eliminate slip in the clamps, linear fixations at the top and bottom were used. prCEN/TS 19102 states additional provisions for testing of ETFE foils regarding the strain rate and prestress level. According to prCEN/TS 19102, the strain rate shall be chosen to $\dot{\varepsilon }$ = 200 (%/min) (termed ${\dot{\varepsilon }}_{200}$ in the following). This strain rate can be compared to the above defined traverse travel speed of ${\dot{v}}_{100}$ (mm/min), when the initial measuring distance equals L_0_ = 50 mm. The initial prestress was set to σ_0_ = 0.5 MPa.

Related to the initial measuring length of L_0_ = 50 mm, the position-controlled tests can be compared to strain-controlled tests as follows:

- ${\dot{v}}_{10}={\dot{\varepsilon }}_{20}$ = 20%/min,
- ${\dot{v}}_{50}={\dot{\varepsilon }}_{100}$ = 100%/min,
- ${\dot{v}}_{100}={\dot{\varepsilon }}_{200}$ = 200%/min,
- ${\dot{v}}_{200}={\dot{\varepsilon }}_{400}$ = 400%/min
- ${\dot{v}}_{500}={\dot{\varepsilon }}_{1000}$ = 1000%/min.

### 2.2. Experimental Procedure and Evaluation Process

Each specimen was prepared following EN ISO 527-1 [18] and -3 [24]. The actual width and thickness were measured. The mean width b_mean_ and mean thickness d_mean_ of each specimen were used for the evaluation of the tensile tests for determination of the resulting stresses and strains. In the tests, the measurements of the force F and the displacement of the traverse L were recorded with a frequency of 10 Hz. The engineering stress σ_eng_ and engineering strain ε_eng_ were calculated by Equations (1) and (2) as followed:

(1) $$\sigma_{\mathrm{eng}}=\frac{F}{b_{\mathrm{mean}}d_{\mathrm{mean}}}$$

and

(2) $$\varepsilon_{\mathrm{eng}}=\frac{L-L_{0}}{L_{0}}=\frac{\Delta L}{L_{0}}$$

Herein, L_0_ is the initial distance of the measuring field. From the measured stress-strain curves, the following characteristic parameters were derived: stresses at specific strains and the stiffness parameters E_0_ (Young’s modulus) and E_ve_. Additionally, the scatter was determined for each parameter by the standard deviation s and the coefficient of variation COV. For each test series, the mean stress-strain curves including the mean stresses at specific strains f*, standard deviation s and COV were determined by Equations (3)–(5) as followed:

(3) $$f_{*}=\frac{1}{n}\sum_{i=1}^{n}f_{*,i}$$

(4) $$s_{f_{*}}=\sqrt{\frac{1}{n-1}\sum_{i=1}^{n}{\left(f_{*,i}-µ\right)}^{2}}$$

(5) $${\mathrm{COV}}_{f_{*}}=\frac{s_{f_{*}}}{f_{*}}$$

Additionally, 5% fractile curves were determined according to EN 1990 [25] by Equation (6):

(6) $$f_{*,k}=f_{*}-k_{n}s_{f_{*}}$$

Herein, k_n_ is the fractile factor according to Table D 1 of EN 1990 [25], considering an unknown COV.

The stresses at specific strain levels were analysed at 1%, 10%, 50%, 100%, 150%, and 200% strain. The stress at 200% strain was taken as the last strength characteristic, since 200% strain was reached in each test independent of the investigated test combinations. Additionally, component tests on surface welded ETFE foils exhibit their breaking strain at roughly 200% strain as well. Here, the typical characteristic breaking stress is f_k,uw_ = 30 MPa at ~200% strain at T = 23 °C and $\dot{\varepsilon }$ = 200%/min. The strength characteristic f_y_ (yield point) was identified at the point at which a stress-strain gradient smaller than 0.001 was achieved. The point f_ve_ was derived by using the calculated stiffness E_ve_ and by identifying the strain at which the difference in the stress of a straight line with the slope equal to E_ve_ and the measured data becomes larger than |0.05 MPa|. This point marks the beginning of the second linear part of the ETFE foil stress-strain behaviour in uniaxial tensile tests. To identify the inflexion point f_ip_, a gradient was used. Here, f_ip_ was defined as the stress-strain point at which the gradient degrades by 80% of its maximum. The authors empirically determined the value of 80% drop down as that point at which the inflexion point could be sufficiently identified for all investigated testing conditions.

In this contribution, the initial stiffness E_0_ was calculated as a regression line between the 0.3% and 0.5% strain. Limiting the strain interval between 0.3% strain and 0.5% strain ensures the elimination of start-up effects and neglects the beginning of the slightly nonlinear behaviour at the end of the first material stage independent of the applied temperature or strain rate. The stiffness after the first inflexion E_ve_ was determined between 5.0% and 5.2% strain. Again, limiting the strain interval to 5.0% to 5.2% eliminates the nonlinearities in the second material stage of the stress-strain behaviour. The advantage of using these relatively small strain ranges for the determination of the stiffness of ETFE foils is that they can be applied for each test temperature and strain rate independent of the material producer and foil thickness.

## 3. Experimental Investigations, Results, Analysis and Discussion

Following the conditions described in the chapters above, uniaxial tensile tests on strip specimens made of ETFE foils from three different material producers were performed in the machine direction (MD) at four different temperatures (T_0_ = 0 °C, T_23_ = 23 °C, T_40_ = 40 °C and T_50_ = 50 °C), five different test speeds (${\dot{v}}_{10}$ = 10 mm/min, ${\dot{v}}_{50}$ = 50 mm/min, ${\dot{v}}_{100}$ = 100 mm/min, ${\dot{v}}_{200}$ = 200 mm/min and ${\dot{v}}_{500}$ = 500 mm/min) and two foil thicknesses (100 µm and 250 µm). For each parameter combination (temperature-test speed-foil thickness), five tests were carried out, apart from the tests at testing temperature T_0_ for the ETFE foil from producer III with only three tests per test combination. In total, 580 uniaxial tensile tests were carried out. Considering measurement uncertainty, all results were rounded to one digit.

All resulting experimental uniaxial stress-strain curves showed the already mentioned three basic parts as expected: the viscoelastic, viscoelastic-plastic and viscoplastic material behaviour with hardening effects in the viscoplastic material stage after yielding. Hereby, the strength point f_ve_ marks the end of the viscoelastic stage and simultaneously the beginning of the second linear part in the viscoelastic-plastic stage. The yield point f_y_, as described in EN ISO 527-1, marks the end of the viscoelastic-plastic stage and simultaneously the beginning of the viscoplastic material stage.

**Figure 3:**
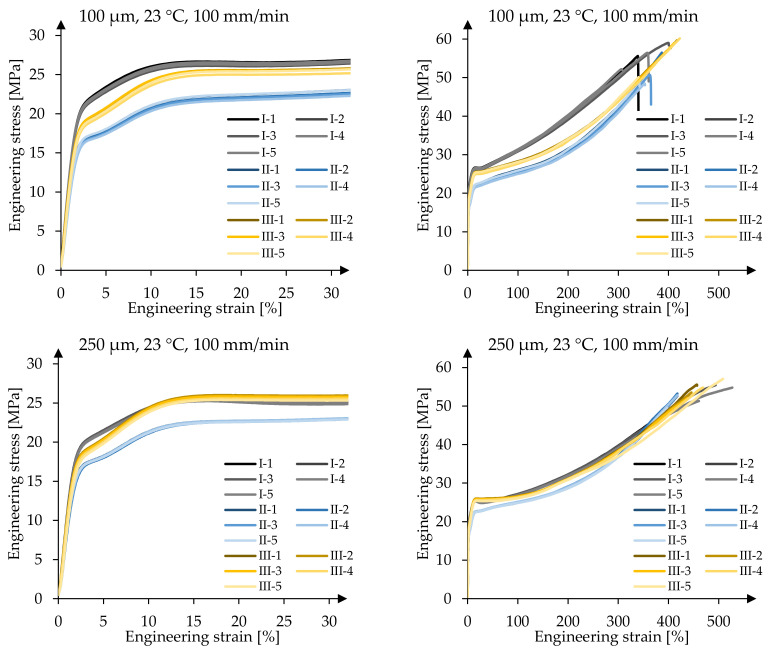
Comparison of uniaxial tensile tests in MD at 23 °C and 200%/min for producers I, II, and III (**left** side diagrams are cut-outs from the **right** side diagrams).

At first, the achieved stress-strain curves at T_23_ = 23 °C and ${\dot{v}}_{100}$ were compared for both foil thicknesses of all three producers, see Figure 3.

The stress-strain curves for each test series show a consistent behaviour with only very small deviations. The resulting coefficients of variation (COV) determined at the previously mentioned specific strains are listed in Table 3. Here, the elastic capabilities are described by the stress f_1_ at 1% strain. The second material stage—the viscoelastic-plastic part—is described by f_10_. The foil producers typically evaluate and issue the stresses at 10% strain as mean values in their data sheets and—if available—in their inspection certificates 3.1 acc. to EN 10204 [26]. Additionally, f_y_ and further stresses were evaluated, the latter ones at specific strains ranging from 50% to 200% strain to characterise the viscoplastic material stage.

In all test series listed in Table 3, the highest COV values occur at the breaking stress, ranging from 3.5% to 7.2%. The main reason for these high deviations is that the breaking stress is not reached at equal strains, but at different strains for each test. This behaviour is explained by the fact that various parameters influence the breaking stress and breaking strain. These parameters are the specimen preparation and the clamping, as well as the clamp itself. On the other side, it becomes obvious that the stress-strain curves for each parameter combination show a very similar behaviour up to 200% strain, and even above. Stresses at 200% strain will not be reached in building structures due to the applied deformation limits in the serviceability limit state. Typically, strains of up to 8% (stress ratio MD:TD = 1:1) to 10% (2:1) at room temperature can be expected in executed ETFE structures when they are designed using the method based on biaxial hysteresis tests offered by prCEN/TS 19102, Annex E (with MD: machine direction and TD: transverse direction). At 200% strain, the highest achieved COV values equal only 1.7% for 100 µm foils and only 0.6% for 250 µm foils. The stresses at the distinct strain values exhibit a very low scatter with COV values up to 1.7%, apart from the scatter at 1% strain. Due to the relatively small stresses at 1% strain of e.g., 10.2 MPa (producer III, 250 µm) with a COV of 6.5%, the standard deviation s yields to 0.7 MPa. However, the measured stress-strain curves in Figure 3 emphasize the overall small scatter, even at 1% strain. In everyday engineering of ETFE foils, the elastic limit represents an important design value, if plastic strains are prohibited in the structure, so that the knowledge of the material behaviour of ETFE foils in the elastic and viscoelastic region is of great interest.

Apart from the overall comparable stress-strain curves for the 250 µm foils, which are given in Figure 3, the mechanical behaviour deviates in the first and second material stages. Here, an influence of the different producers can be observed. Tests carried out at 250 µm foils of producers I and III result in a similar stress-strain behaviour in the elastic up to the end of the viscoelastic region, represented by f_1_ and f_ip_, respectively, the viscoelastic-plastic stage, characterised by f_10_ as well as the yield point f_y_, and the stress at 200% strain f_200_, which describes the viscoplastic stage. In contrast, the material of producer II exhibits considerable smaller stresses at above mentioned strains compared to those materials of the other producers. This behaviour can be observed for both foil thicknesses. For example, the mean f_1_-values for the 250 µm foils are 10.8 MPa and 10.2 MPa for producers I and III, and 9.2 MPa for producer II. However, the 100 µm foils show different behaviours in all three material stages and for all three producers. Here, the stresses at 1% strain equal 12.6 MPa, 11.3 MPa and 10.0 MPa for material producers I, III, and II, respectively. Even though the absolute deviations at 1% strain across the investigated foils of the three producers seem quite small with values of Δ_250µm,I–II_ = 1.6 MPa for the 250 µm foils and Δ_100µm,I–II_ = 2.6 MPa for the 100 µm foils, the percentile increase from producer II to I equals 15.7% and 26.0%, respectively. This results in considerably different design limits for the serviceability limit state design (SLS) used in prCEN/TS 19102 which should not be neglected for economic reasons. Currently, in the SLS design, the characteristic elastic stress limit at room temperature f_el23_ can be taken as 15 MPa according to prCEN/TS 19102. f_el23_ can be compared to f_ip_. The experimental results show that the characteristic elastic stress limit f_el23_ complies with the proposed value of 15 MPa and that significantly higher design limits depending on the used material can be achieved. For both foil thicknesses, the material producer II displays overall lower stresses at specific strains than the material of the other two producers. Additionally, it becomes obvious that 100 µm foils achieve generally higher stress levels at specific strains compared to 250 µm foils.

**Figure 4:**
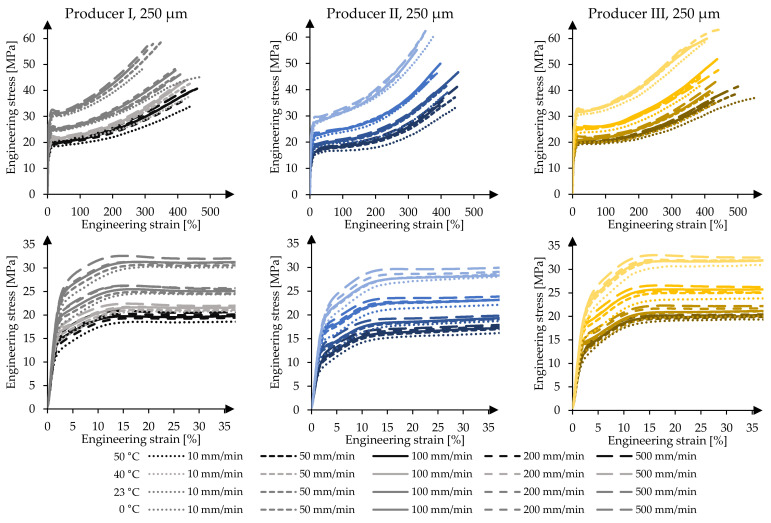
Mechanical response of 250 µm ETFE foils in uniaxial tensile tests at varying test temperatures and test speeds as mean value curves per test series, for materials of producers I, II, and III, respectively.

To illustrate the temperature- and strain rate-dependent material behaviour of the 250 µm foils, the mean value stress-strain curves of the uniaxial tensile tests are displayed in Figure 4. With a decrease in temperature, the material’s strength increases. An equivalent material response occurs with an increase in the strain rate. A contradictory material behaviour is present if the temperature increases and/or the strain rate decreases as the ETFE foil gets softer and weaker. The stress-strain curves show that the test temperature has a significantly higher influence on the material behaviour than the strain rate. For instance, the 10%-strain stress can vary by a factor of roughly 1.6 for producers I and III and 1.7 for producer II in the tested temperature range. In the tested range of strain rates, it varies only by a factor of about 1.15 for all three producers. Furthermore, with a decrease in temperature, the elongation capability of the material is reduced. Simultaneously, the achievable breaking stresses increase. Equivalent material behaviour is also described in, e.g., [7,8,9,10,12,13,14,15,16,17].

The temperature and strain rate also influence the yielding capability of ETFE foils. The lower the test temperature and the higher the strain rate, the smaller the elongation capability. Using the definition of the yield point of EN ISO 527-1, it can be observed that the yield point f_y_ for material of producer II is not clearly delineated in tests performed with low strain rates. This behaviour changes with an increase of the strain rate. Under comparatively high strain rates, distinct yield points become visible. The materials of producers I and III, on the other hand, exhibit clearly determinable yield points with upper and lower bounds, even at high temperatures and low strain rates. Here, after reaching the yield point f_y_, a decrease of the stress is measurable even though the strain increases (see Figure 4). These findings illustrate that the presence of a clear yield point is not a characteristic of ETFE in general but depends on the recipe or the manufacturing conditions. Additionally, it emphasises that ETFE behaves differently depending on the producer. Despite the fact that a clearly determinable yield point is not present for every test combination, the material stiffness for every foil product (I, II or III) drastically changes with reaching the third material stage. In uniaxial tensile tests, yielding—beginning of material stage three—occurs between 12% and 20% strain depending on the test combination of temperature, strain rate, and material.

**Figure 5:**
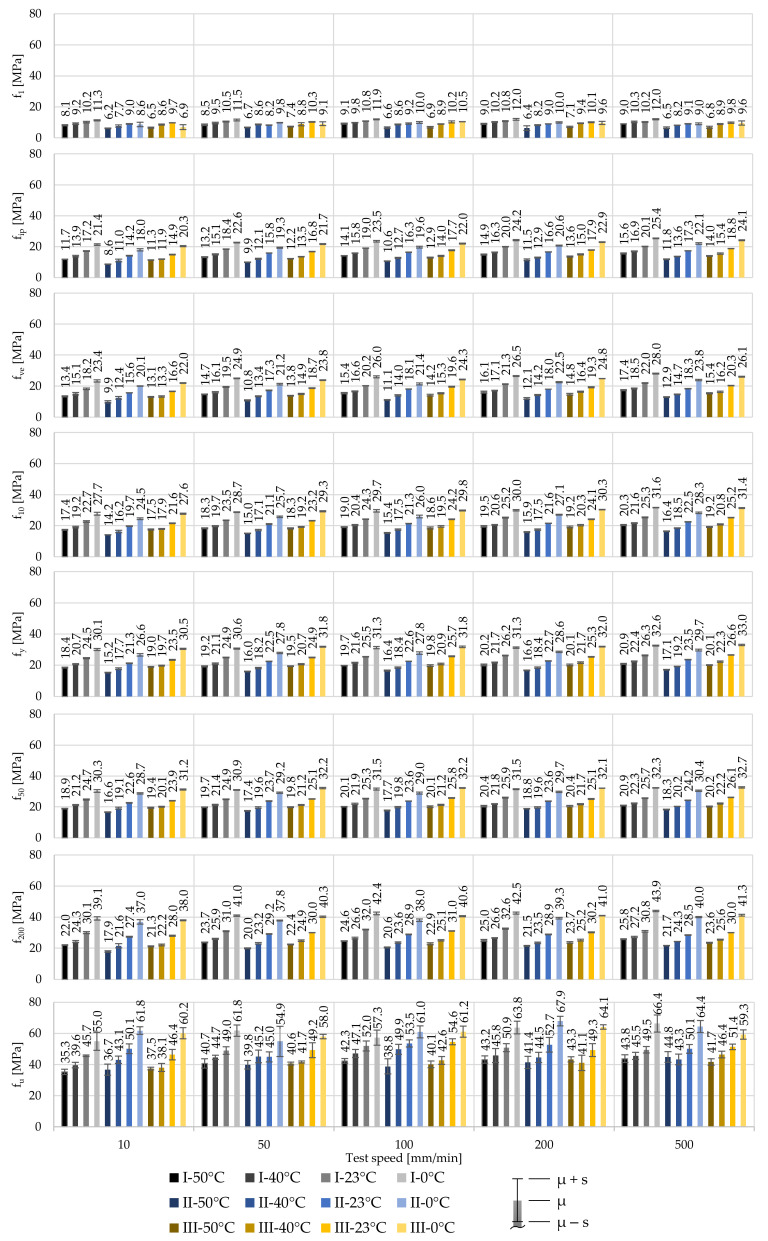
Influence of the test temperature and test speed on the stress at specific strains for 250 µm ETFE foils of producers I, II, and III as mean values.

Evaluating the illustrated uniaxial tensile tests and following the determination process described in chapter 2.2 regarding the stress parameters f*, as well as the stiffness parameters E_0_ and E_ve_, allows for insight into the material behaviour and its characterisation process. To design foil structures, knowledge about the elastic stage, as well as the corresponding stiffness E_0_, is required. Here, the proportional limit is described by f_1_. Figure 5 contains the derived mean stresses f* for 250 µm foils at specific strains, 1%, 10%, 50% and 200%. Additionally, values are given for the first inflexion point f_ip_, the strength characteristic f_ve_, the yield stress f_y_ and the ultimate tensile strength f_u_. The standard deviations s are illustrated using whiskers.

All derived stresses emphasise the temperature- and strain rate-dependent material behaviour in all three material stages (viscoelastic, viscoelastic-plastic, viscoplastic). The influence of the temperature and strain rate increases with an increase of the applied stresses. Exemplary, focusing on the stresses at 1% strain shows the relatively low impact of the test speed. Here, at T_23_, an increase of the test speed from 10 mm/min to 500 mm/min (factor 50) leads to an increase of the stresses of Δ_I,f1,v500–v10_ = 0 MPa (0%), Δ_II,f1,v500–v10_ = 0.1 MPa (1.5%) and Δ_III,f1,v500–v10_ = 0.1 MPa (1.2%), for producers I, II, and III, respectively, with their low COVs ranging from 1.3% to 4.7% at the investigated test combinations. This means that influence of the test speed and thus the strain rate is rather small in the proportional region. Determining the impact of the test speed at f_10_, the same increase of the test speed from 10 mm/min to 500 mm/min yields to increases in the stresses of Δ_I,f10,v500–v10_ = 2.6 MPa (11.5%), Δ_II,f10,v500–v10_ = 2.8 MPa (14.2%) and Δ_III,f10,v500–v10_ = 3.7 MPa (17.0%), respectively. At f_10_ and at these investigated test combinations, the COVs range from only 0.4% to 2.4%, which suggests a constant mechanical behaviour independent of the test combination. Apparently, the impact of increasing strain rates is linked to the first inflexion point f_ip_. Here, the same increase of the test speed leads to increases of the stress of Δ_I,fip,v500–v10_ = 3.0 MPa (17.3%), Δ_II,fip,v500–v10_ = 3.1 MPa (22.1%) and Δ_III,fip,v500–v10_ = 3.9 MPa (26.0%), respectively. In contrast, the effects of different test temperatures are comparatively high. An increase of the test temperature from 0 °C to 50 °C (± 2 K) at a test speed of ${\dot{v}}_{500}$ results in deviations of the stresses at 1% strain of Δ_I,f1,T50–T0_ = −3.0 MPa (–24.9%), Δ_II,f1,T50–T0_ = −2.5 MPa (−27.3%) and Δ_III,f1,T50–T0_ = −2.8 MPa (−29.5%), respectively. This influence even increases at f_10_ to deviations of Δ_I,f10,T50–T0_ = −11.3 MPa (−35.7%), Δ_II,f10,T50–T0_ = −11.9 MPa (−42.1%) and Δ_III,f10,T50–T0_ = −12.2 MPa (−38.9%), respectively. At f_1_ the COVs range from 2.0% to 14.5% and at f_10_ from only 0.6% to 1.9%. The relatively high scatter of 14.5% equals a standard deviation of 1.4 MPa and occurs at T_0_ for producer III. However, the small COVs at 10% strain emphasize the constant material behaviour.

These results illustrate the nonlinear degressive temperature and progressive strain rate dependencies on the strength of ETFE foils. Shortly after reaching 10% strain, the yield point f_y_ occurs. Again, the mechanical responses of the materials of producers I and III are similar. Here, at T_23_ and ${\dot{v}}_{10}$, yielding occurs at comparable stresses of 24.5 MPa and 23.5 MPa for materials of producers I and III with very low COVs of 1.6% and 0.9%, respectively. The material of producer II exhibits smaller stresses at comparable strains (e.g., yielding occurs at 21.3 MPa under same temperature and test speed conditions but with also a very low COV of 0.9%). By comparing the stresses at f_y_ and f_50_, it becomes clear that in buildings structures yielding of the ETFE foils has to be for sure avoided. By reaching higher stresses than f_y_, a structural displacement control is not ensured anymore. Under high strains occurring after yielding (e.g., due to water ponding), events or failure due to contact with other components may occur.

Additional information can be achieved by evaluating the standard deviation of each test combination, illustrated by the whiskers in the diagrams. Here, independent of the different materials investigated, different test temperatures and test speeds do not show any influence on the achieved standard deviations for all test series. Herewith, it can be seen, that ETFE foils demonstrate a constant mechanical behaviour in short-term uniaxial tensile tests. However, the derived standard deviations at f_u_ exhibit a relatively high scatter in the stresses, ranging from COV = 1.0% to 17.6%. In addition to the comparatively high scatter of the breaking stresses, different breaking strains occur as well. This makes a comparison of different breaking stresses difficult. As mentioned above and to characterise the viscoplastic stage, f_200_ has been introduced in the presented study. Even at 200% strain, the materials of producers I and III show higher strengths than the material of producer II, e.g., with 30.1 MPa, 27.4 MPa, and 28.0 MPa for producers I, II, and III at T_23_ and ${\dot{v}}_{10}$, respectively. Their COVs are very low, ranging from 2.2% (producer I) to 0.9% (producer II).

**Figure 6:**
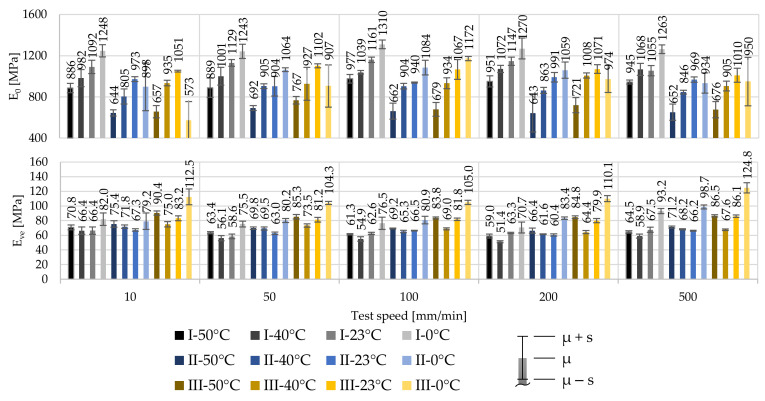
Influence of the test temperature and test speed on E_0_ and E_ve_ for 250 µm ETFE foils of producers I, II, and III.

The relative high influence of the test temperature and comparatively low influence of the strain rate can also be observed when looking at the material stiffnesses. An increase in the strain rate does not increase the stiffness significantly compared to the impact of a reduction in test temperature. With a decrease of the test temperature, the stiffness increases. However, irregularities in the stress at 1% strain and in the Young’s modulus can be observed, both, in the influence of test temperatures and strain rates. The derived initial material stiffnesses E_0_ are illustrated in Figure 6. It can be observed that material of producer I shows a distinctly stiffer material behaviour than material supplied by producers II and III, independent of the test condition. Comparing the material response of the material of producers II and III, similarities are recognized. This follows the observations already made for f_1_. By reaching the viscoelastic-plastic material stage after the first inflexion point, the material stiffness drastically decreases independent of the test combination or material. The material of producer III indicates the stiffest material response after the first inflexion point.

**Figure 7:**
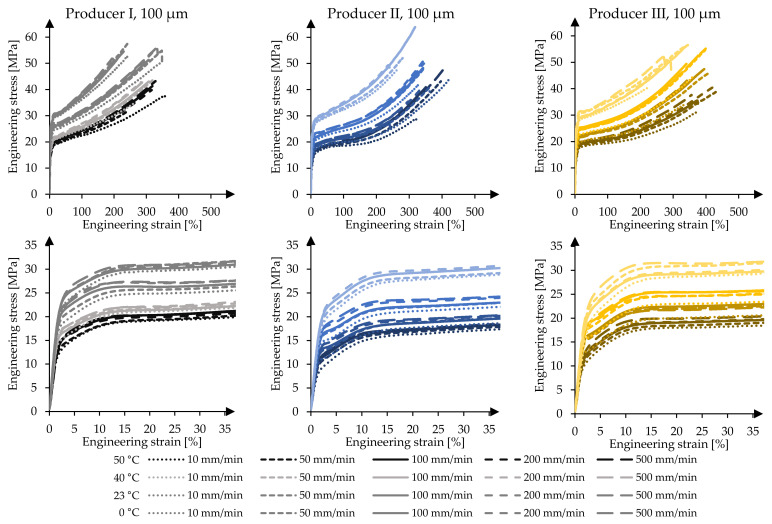
Mechanical response of 100 µm ETFE foils in uniaxial tensile tests at varying test temperatures and test speeds as mean value curves per test series, materials of producers I, II, and III, respectively.

Figure 7 illustrates the material responses of the 100 µm foils. As for the 250 µm foils, the 100 µm foils show a similar temperature and strain rate dependent mechanical behaviour. With a decrease of the temperature and an increase of the strain rate, the material’s strength increases independent of the producer. Regarding the breaking strains, the 100 µm foils show a smaller elongation capability than the 250 µm foils. This complies with a smaller yield plateau achieved by the 100 µm foils compared to that achieved by the 250 µm foils. A distinct and long yield plateau cannot be identified due to the fact that the stress and strain constantly increase. Additionally, the material of producer II shows again smaller stresses at specific strains compared to the foils of the other two material producers. A remarkable influence caused by the material thickness can be observed in material stage three after yielding. Shortly after reaching f_y_, the hardening characteristics can again be identified.

**Figure 8:**
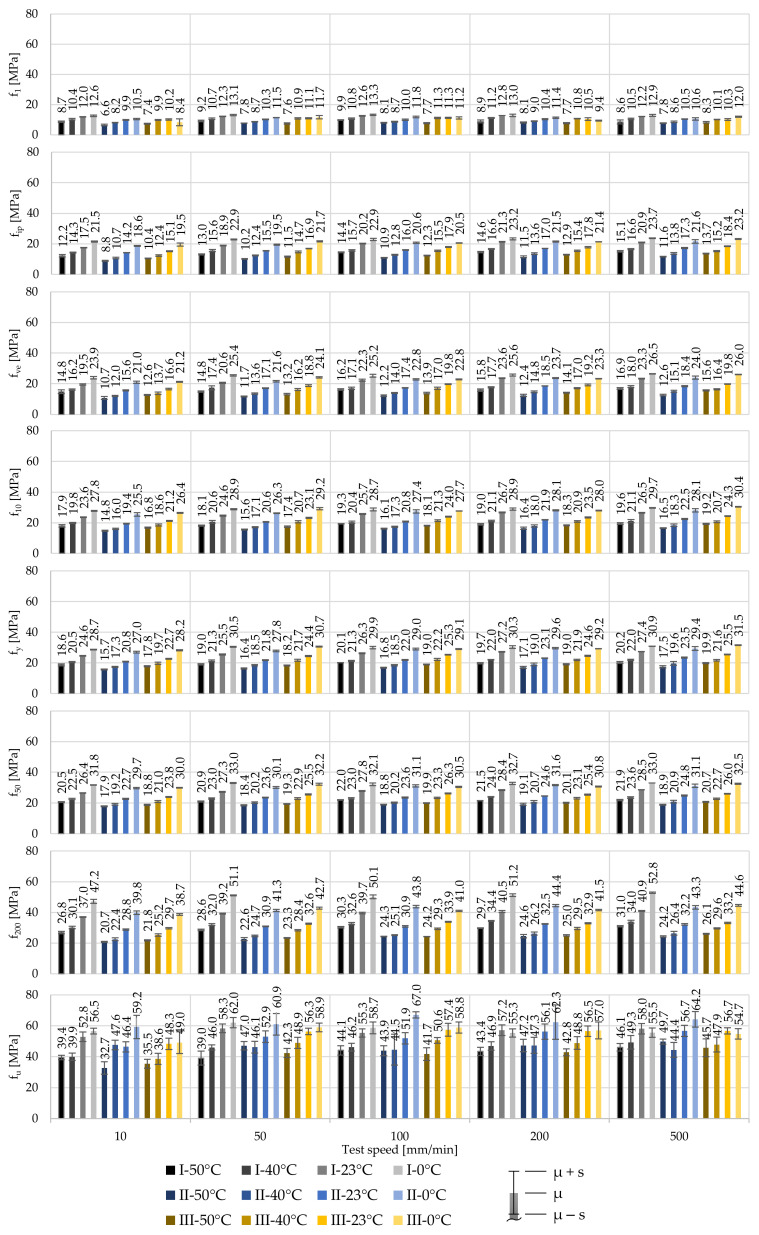
Influence of the test temperature and test speed on the stress at specific strains for 100 µm ETFE foils of producers I, II, and III.

Figure 8 illustrates the mean stresses at specific strains for the 100 µm and 250 µm foils under the influence of varying test temperatures and test speeds. As in the case of the 250 µm foils, with decreasing temperatures and increasing strain rates, the stresses of the 100 µm foils at comparable strains increase. Again, the response caused by increasing the strain rates at constant test temperature is less significant compared to the impact of changing test temperatures and keeping the strain rate constant. This can be shown using the derived f_1_-values at T_23_ for test speeds ${\dot{v}}_{10}$ and ${\dot{v}}_{500}$. Increasing the test speed by a factor of 50 leads to an increase of the stress at 1% strain of Δ_I,f1,v500–v10_ = 0.2 MPa (1.6%), Δ_II,f1,v500–v10_ = 0.6 MPa (5.9%) and Δ_III,f1,v500–v10_ = 0.1 MPa (0.6%), for materials of producers I, II and III, respectively. Here, the COVs range from low 1.2% to moderate 5.9%. Analysing the stresses at 10% strain f_10_ shows that the same increase of the test speed—10 mm/min to 500 mm/min—leads to increased stresses of Δ_I,f10,v500–v10_ = 3.0 MPa (12.5%), Δ_II,f10,v500–v10_ = 3.0 MPa (15.6%) and Δ_III,f10,v500–v10_ = 3.1 MPa (14.4%) with COVs ranging from only 0.7% to 1.5%. The material thickness affects the stress levels in general. For 100 µm foils, the overall measured stresses at comparable strains are higher than those achieved for 250 µm foils. For example, at 1% strain, the 100 µm foils of producer I exhibit 12.6 MPa at T_23_ and ${\dot{v}}_{100}$ with a COV of 1.1% and the 250 µm foils exhibit 10.8 MPa with a COV of 1.6%. This indicates higher stiffnesses for 100 µm foils as well.

**Figure 9:**
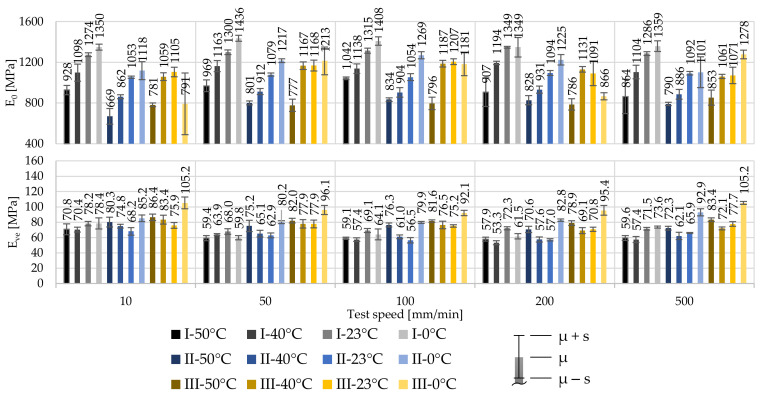
Influence of the test temperature and test speed on E_0_ and E_ve_ for 100 µm ETFE foils of producers I, II, and III.

The derived material stiffnesses for the first and second material stages are illustrated in Figure 9. As for 250 µm foils, the material stiffness of the 100 µm foils increases significantly with decreasing test temperatures. The impact of the strain rate, however, is again far less significant.

Comparing the stiffnesses of the 250 µm foils with the derived stiffnesses of the 100 µm foils underlines the achieved slightly higher stresses at f_1_ of 100 µm. For example, the stiffnesses E_0_ at T_23_ and ${\dot{v}}_{100}$ are E_0,I,250µm_ = 1161 MPa, E_0,II,250µm_ = 940 MPa and E_0,III,250µm_ = 1067 MPa with COVs of 2.2%, 0.6% and 9.1% for materials of producers I, II and III, respectively. In contrast, the stiffnesses for 100 µm foils at T_23_ and ${\dot{v}}_{100}$ become E_0,I,100µm_ = 1315 MPa, E_0,II,100µm_ = 1054 MPa and E_0,II,100µm_ = 1207 MPa with COVs of 1.9%, 3.2% and 2.4%, respectively. Herewith, a decrease of the foil thickness of 40% leads to an increase of the stiffness of about 13.3%, 12.1%, and 13.1% for this specific test combination, respectively.

Nevertheless, comparing the first stage and the second stage of the measured stress-strain curves for the 100 µm foils and 250 µm foils, the materials show similar responses, so that an analytical model can be developed to describe the stress-strain behaviour of ETFE foils under consideration of temperature and strain rate effects. To cover the ETFE foil market for both foil thicknesses, 5% fractile curves were derived independently of the material producer but depending on the foil thickness.

## 4. Analytical Modelling of the Temperature and Strain Rate Dependent Material Behaviour

### 4.1. General

To simulate the temperature and strain rate dependent material behaviour of ETFE foils, a modified Ramberg-Osgood-Material model is introduced. Nowadays, various modified Ramberg-Osgood-Material models are widely used. The original model derived by W. Ramberg and W.R. Osgood is based on three parameters considering the Young’s modulus and two secant yield strengths [19]. On the basis of the Ramberg-Osgood-model, first Rasmussen [20] and afterwards Arrayago, Real and Gardner [21], see also [22], developed a modified material for describing the stress-strain behaviour of stainless steel using additional parameters K, n and m, for describing the nonlinear stress-strain relationship. As the stress-strain behaviour of stainless steel exhibits a quite similar course of the stress-strain curve as ETFE foils show, it was decided to rely on the modified material model by Arrayago, Real and Gardner [21] for developing an analytical material model for ETFE foils. In the following, first the modified material model according to Arrayago, Real and Gardner [21] is described shortly. Originally, for stainless steel, the yield point f_y_ and the ultimate tensile strength f_u_ were used, which also describe the limits of the individual formulas. To describe the elastic stage of the nonlinear material (e.g., stainless steel), Equation (7) is introduced, which is limited by the yield strength, f_y_:

(7) $$\varepsilon =\frac{\sigma }{E}+K{\left(\frac{\sigma }{f_{y}}\right)}^{n}\;\mathrm{for}\;\sigma \;\leq \;f_{y}$$

Here, ε is the calculated strain, σ the applied stress, E the Young’s modulus and n and K model parameters. To model the strain after yielding, Equation (8) is used:

(8) $$\varepsilon =K+\frac{f_{y}}{E}+\frac{\sigma -f_{y}}{E_{y}}+\varepsilon_{u}{\left(\frac{\sigma -f_{y}}{f_{u}-f_{y}}\right)}^{m}\;\mathrm{for}\;f_{y}\;<\;\sigma \;\leq \;f_{u}$$

**Figure 10:**
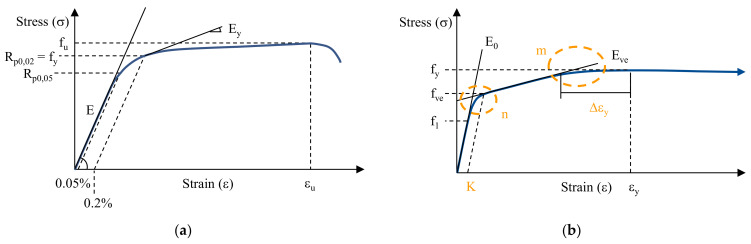
Stylized stress-strain curve of (**a**) stainless steel with Ramberg-Osgood parameters, following [22] and (**b**) ETFE foils with modified Ramberg-Osgood parameters.

In this formula, three additional parameters are integrated: model parameter m to describe the nonlinearity, the ultimate tensile strength f_u_, and the corresponding strain ε_u_. Figure 10a illustrates the parameters used to describe a stress-strain path for stainless steel.

Adjusting this model to describe the uniaxial stress-strain behaviour of a short-term tensile test of ETFE foils, the parameter f_y_ for stainless steel is modified to f_ve_. Herewith, Equation (7) describes the first stage of ETFE foils in a uniaxial tensile test. To describe the second stage, the parameters in Equation (8) are modified to f_ve_, f_y_ and Δε_y_, respectively. Implementing the described parameters, the modified Ramberg-Osgood model for ETFE foils can be given by Equations (9) and (10), see also Figure 10b:

(9) $$\varepsilon =\frac{\sigma }{E_{0}}+K{\left(\frac{\sigma }{f_{ve}}\right)}^{n}\;\mathrm{for}\;\sigma \;\leq \;f_{\mathrm{ve}}$$

(10) $$\varepsilon =K+\frac{f_{\mathrm{ve}}}{E}+\frac{\sigma -f_{\mathrm{ve}}}{E_{\mathrm{ve}}}+\Delta \varepsilon_{y}{\left(\frac{\sigma -f_{\mathrm{ve}}}{f_{y}-f_{\mathrm{ve}}}\right)}^{m}\;\mathrm{for}\;f_{\mathrm{ve}}\;<\;\sigma \;\leq \;f_{y}$$

Comparing the two stylized stress-strain curves for stainless steel and ETFE foils in Figure 10a,b the different approaches become obvious which have to be applied for modelling the stress-strain behaviour in the second material stage after the first inflexion point. For this reason, for ETFE-foils, the changing point in the formulas is set to a different material characteristic. For stainless steel, the yield point f_y_ is described as R_p0.2_, which is defined by the stress resulting in 0.2% strain after unloading. Simultaneously, at this point, the yield modulus E_y_ is calculated. Herewith, the nonlinearity at the yield point and the linearity in the hardening can be modelled. However, for ETFE foils, the second material stage exhibits two nonlinearities (the first shortly after the first inflexion point and the second close to the yield point). To model this material behaviour, the formula-changing point is set to f_ve_, which marks the beginning of the second linear material response in a uniaxial tensile test of ETFE foils. Herewith, the two occurring nonlinearities can be described analytically. To derive the interchange point, the modulus E_ve_ determined in the second material stage is used. As described in Section 2.2, the modulus E_ve_ is calculated using the strain interval 5.0% to 5.2% by regression analysis. Formulating a straight line with E_ve_ as its slope, the point f_ve_ is identified at which the measured stress-strain deviates more than 0.05 MPa at equal strains.

### 4.2. Evaluation of the Model Parameters

To determine the stress limits where the function changes—f_ve_ and f_y_—while considering the temperature and strain rate dependent behaviour leads to the following Equation (11):

(11) $$f_{*}=\exp\left(a+\frac{b}{\dot{v}+c}\right)\left(d\left(T+T_{\mathrm{ref}}\right)+f\right)$$

Herein, a, b, c, d, and f are further model parameters, determined using R^2^ and the Levenberg-Marquardt algorithm [27,28]. T_ref_ is the reference temperature of −296.15 K. T and $\dot{v}$ describe the test temperature (K) and test speed (mm/min]). The term regarding the influence of test speeds follows the research carried out in reference [9]. Using the established function, in the first step, the model parameters can be determined for each producer and foil thickness. Additionally, the first inflexion point f_ip_ can be calculated as well. However, to represent ETFE foils, which are present on the market, parameters have to be determined matching calculated 5% fractile stress-strain curves and their characteristic values, independent of the material producer. Here, designers can predict the mechanical material behaviour under uniaxial tension for ETFE foils depending on the service temperature on site or the strain rate of the investigated load scenario (e.g., load combinations regarding snow or summer storms). The 5% fractile stress-strain curves were determined according to EN 1990 [25]. Table 4 summarises the derived model parameter for Equation (11). Their fits are illustrated by R^2^. Of course, further generalizing of the parameters will be required in order to simplify the usage for practical design.

As an example, the model predicts the yield stress f_y_ for an ETFE foil of 250 µm at a test speed of $\dot{v}$ = 10 mm/min at T = −23 °C = 250.15 K to

(12) $$f_{k,y,250µm,250.15K,10\mathrm{mm}/\min}=30.48\;\mathrm{MPa}$$

To validate the predicted yield stress, tensile tests at the previously mentioned test combination were performed for materials of producers I and II. The measured 5% fractile yield stresses for these materials equal f_k,y,I,250µm,250.15K,10mm/min_ = 34.56 MPa and f_y,II,250µm,250.15K,10mm/min_ = 31.86 MPa. The predicted yield stress at T_−23_ = 30.48 MPa complies conservatively with the measured 5% fractile values of the measured yield points.

The necessary model parameters to predict the uniaxial material behaviour of ETFE foils—K, n, m and Δε_y_—were determined. As for Equation (11), the model parameters were derived depending on the foil thickness, but not on the material producer. Table 5 lists generalized model parameters depending on the applied temperature but not on the test speed and thus strain rate. Further generalizing of the model parameters will be needed in order to simplify the usage of the proposed material model for structural engineers and its application in standardization work. For this and for further simplification of Equations (9)–(11), additional and exemplary uniaxial tensile tests will be carried out for a third foil thickness.

### 4.3. Prediction of the Mechanical Material Behaviour of ETFE Foils under Uniaxial Tension

**Figure 11:**
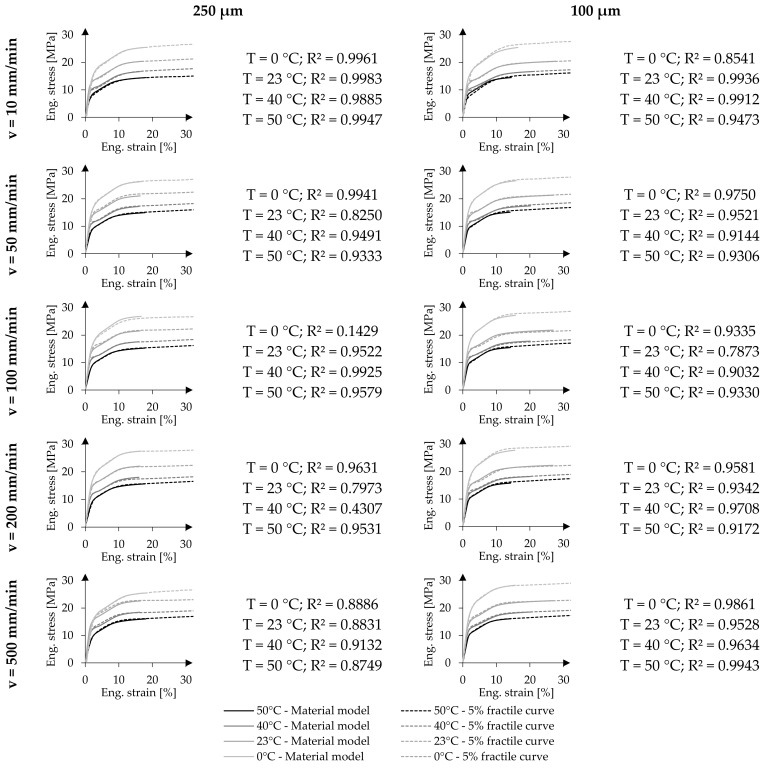
Predicted uniaxial material behaviour of 250 µm and 100 µm ETFE foils under uniaxial tension at varying test temperatures and strain rate with their comparison to 5% fractile curves.

Using the above presented model parameters, uniaxial tensile stress-strain curves can be calculated. Figure 11 visualizes the predictions in comparison to the 5% fractile curves at the investigated test temperatures and strain rates for the two nominal foil thicknesses 250 µm and 100 µm, respectively. Each diagram illustrates a cutout up to 30% strain and 30 MPa stress. Herewith, the diagrams contain the individual inflexion points f_ip_ and yield points f_y_. The quality of the fit is provided by R^2^ describing the overall curve fit in each test combination. Here, R^2^ relates to the predicted strain at specific stress levels.

Focusing on the curves of 250 µm ETFE foils, R^2^ varies between 0.9983 and 0.7973. However, two extraordinary low R^2^-values are obtained, for the test combinations T_0_ ⊕ ${\dot{v}}_{100}$ and T_40_ ⊕ ${\dot{v}}_{200}$ with R^2^ = 0.1456 and R^2^ = 0.4307, respectively. In both cases, the established material model with generalized model parameters predicts lower strain values than measured strain values at equal stresses. These relatively poor R^2^-values result from the combination of slightly too low parameters Δε_y_ determining the yield strain. By underestimating the yield strain, the deviation between the model and the 5% fractile stress-strain curve explains the poor R^2^, since a slight increase in stress results in high strain deviations. Of course, the fit can be optimised by individually determining the model parameters K, n, m and Δε_y_. For the test combination T_0_ ⊕ ${\dot{v}}_{100}$ the fit can be improved to R^2^ = 0.9939 by shifting Δε_y_ = 0.05 to Δε_y_ = 0.25. For the other problematic test combination (T_40_ ⊕ ${\dot{v}}_{200}$), Δε_y_ can be shifted from Δε_y_ = 0.05 to Δε_y_ = 0.20 to increase R^2^ = 0.9917. The other predicted uniaxial material behaviour of 250 µm foils show good alignments with the measured 5% fractile curves independent of the material producer. For the 250 µm foils, the uniaxial mechanical response under different test temperatures and strain rates of the materials available on the market can be predicted up to the yield point. Additionally, the strain at f_ve_ and the yield point f_y_ can be calculated.

Reviewing the predictions regarding the material behaviour of 100 µm ETFE foils in comparison to the 5% fractile curves, similarly good fits are present, which are illustrated in Figure 11 as well. Here, the predictions and their coefficients of determination R^2^ range between 0.9943 and 0.7873. This suggests a generally good fit and a decent representation of the uniaxial material behaviour of 100 µm foils up to the yield point using the generalised model parameters given in Table 5. However, the proposed material model and its strain vastly depend on the predicted characteristic stress points f_ve_ and f_y_, indicating a change in the material behaviour from viscoelastic to viscoelastic-plastic and eventually to viscoplastic.

For further description of the model’s fit, calculated strains using the material model at specific stresses are compared to the calculated 5% fractile stress-strain curves of the experimental data (see Table 6). Here, exemplary test conditions of low temperatures with comparatively low strain rates and high temperatures with comparatively high strain rates are shown to represent slow growth of snow loads and fast growth of wind gusts, respectively. Additionally, the calculated and measured strains under the laboratory test combination T_23_ ⊕ ${\dot{\varepsilon }}_{200}$(≅${\dot{v}}_{100}$) as described in [4] are listed in Table 6. For each test combination the strain values at 4 MPa (typical prestress level), f_el23_ = 15 MPa and 20 MPa are issued. The absolute strain differences (Δε = ε_calc_ − ε_measured_) between the model and 5% fractiles are given.

Using the generalized model parameters listed in Table 5, the calculated Δε in Table 6 emphasize the overall good fit with the lowest deviation of 0% strain and the highest deviation of 0.9% strain both at T_50_ ⊕ ${\dot{v}}_{500}$. However, deviations can be on either side, positive and negative. When applying the generalized model parameters, the quality of the individual fits naturally scatters. However, by fitting the 5% fractile stress-strain curves from uniaxial tensile tests of the materials of three different producers, the ETFE foils available on the market are represented for the investigated foil thicknesses of 100 µm and 250 µm. To evaluate the influence of the derived analytical model in comparison to experimental results, an imaginary 1-dimensional ETFE foil of 4 m span width is modelled and the sag is evaluated depending on the different strains ε_calc_ and ε_measured_ for the test combination of T_23_ ⊕ ${\dot{v}}_{100}$ at 20 MPa. For a 100 µm ETFE foil, with ε_calc_ = 9.4% strain the sag equals 0.78 m; with ε_measured_ = 9.1% strain the sag equals 0.77 m. For a 250 µm ETFE foil, with ε_calc_ = 10.6% strain the sag is 0.83 m; with ε_measured_ = 11.5% strain the sag is 0.87 m. These differences are negligible in the context of structural design. This emphasises that the derived analytical material model can be used to predict the structural behaviour of ETFE foils even though strains are over- and underestimated up to ±0.9% strain. Currently, further investigations into covering other foil thicknesses as well as simplification and generalization of the developed material model will be carried out.

## 5. Conclusions

To evaluate the mechanical behaviour of ETFE foils under varying test conditions, uniaxial tensile tests were performed. Herein, four different test temperatures ranging from 0 to 50 °C were investigated. Additionally, five different test speeds and their strain rate effect on the material behaviour were analysed ranging from 10 mm/min (≅20%/min) to 500 mm/min (≅1000%/min). Furthermore, two different foils thicknesses and materials of three different producers were considered in order to represent ETFE foils currently available on the market. First, uniaxial tensile tests were performed considering the mentioned test combinations. Here, the way in which the mechanical response of the ETFE foils influenced by the test temperature and strain rate became clear across the different products. Generally, the material response of thermoplastic ETFE can be described as nonlinear. It can be divided into viscoelastic, viscoelastic-plastic, and viscoplastic deformation stages. Distinctly different material responses can be observed depending on the various material producers and foil thicknesses. The material of producer II showed distinctly higher strains compared to the material of producers I and III at comparable stresses. This material response is independent of the applied test combination or investigated foil thickness. Regardless of the analysed material, the mechanical behaviour under uniaxial tension showed dependencies regarding the applied test speed (and thus strain rate) and test temperature. With an increase of the strain rate, strength and stiffness of all three different materials also increased. However, with an increase of the test temperature, a contrary mechanical response is present. The influence of the test temperature is also significantly higher than the influence of the strain rate.

To resemble and cover ETFE foils available on the market, 5% fractile stress-strain curves per nominal foil thickness and independent of the material producer were calculated for each investigated test combination. To model and predict the mechanical behaviour under uniaxial tension under different strain rates and temperatures, a nonlinear model, based on the modified Ramberg-Osgood-material model, has been established. The derived material model is characterised by an interchange of two dependent functions. Interchanging at the stress point f_ve_, characterised by the beginning of the second linear material part after the first inflexion point in a stress-strain diagram, the mechanical response of ETFE foils can be modelled up to the yield point f_y_. Here, three distinct deformation stages can be determined. Functions to predict the interchanging points f_ve_ and f_y_, which cover the limits of each function, were derived depending on the applied test speed and test temperature. Additionally, a function to predict the first inflexion point f_ip_—commonly called the first yield point—has been established as well. With the established functions, the additional model parameters were calculated and then generalized for each test temperature, to allow easy applicability. With the development of the nonlinear material model, the mechanical behaviour of ETFE foils regardless of the given environmental conditions on site and independent of the material supplier can be predicted. In future, further investigations will be carried out to simplify the analytical material model for ease of use in daily design practice.

## Figures and Tables

**Table 2:** Test matrix for the determination of the influence of different test temperatures and strain rates on ETFE foils.

Producer	Foil Thickness (µm)	Test Direction	Temperature (°C)	Strain Rate (mm/min)
I, II and III	100, 250	MD	0, 23, 40, 50	10, 50, 100, 200, 500
⇒ 3	⇒ 2		⇒ 4	⇒ 5

**Table 3:** Mean stress values per test series at specific characteristics and their COV at T_23_ and ${\dot{v}}_{100}≅{\dot{\varepsilon }}_{200}$.

Foil Thickness	Producer	Parameter	f_1_	f_ip_	f_10_	f_y_	f_50_	f_100_	f_150_	f_200_	σ_b_
100 µm	I	σ_mean_ (MPa)	12.6	20.2	25.7	26.3	27.8	31.1	35.0	39.7	55.3
		COV (%)	1.1	0.9	1.0	1.0	0.8	0.8	0.9	1.2	4.9
	II	σ_mean_ (MPa)	10.0	16.0	20.8	22.0	23.6	25.5	27.6	30.9	51.9
		COV (%)	3.9	1.2	1.5	1.5	1.5	1.7	1.4	1.7	7.1
	III	σ_mean_ (MPa)	11.3	17.9	24.0	25.3	26.3	28.0	30.4	33.9	57.4
		COV (%)	3.4	1.4	1.1	0.98	1.0	0.9	0.8	0.8	7.2
250 µm	I	σ_mean_ (MPa)	10.8	19.0	24.3	25.5	25.4	27.0	29.2	32.1	52.0
		COV (%)	1.6	0.4	0.6	0.6	0.5	0.6	0.6	0.6	6.1
	II	σ_mean_ (MPa)	9.2	16.3	21.3	22.6	23.6	25.0	26.5	28.9	53.5
		COV (%)	4.6	0.5	0.5	0.3	0.3	0.4	0.4	0.6	4.3
	III	σ_mean_ (MPa)	10.2	17.7	24.2	25.1	25.8	26.4	28.1	31.0	54.6
		COV (%)	6.5	1.4	1.0	1.0	1.1	0.9	0.5	0.4	3.5

**Table 4:** Derived characteristic model parameters describing the temperature and strain rate dependence of f_ve_ and f_y_ (MPa) of ETFE foils.

Foil Thickness	Parameter	a	b	c	d	f	R^2^
100 µm	f_k,ip_	1.981	−13.61	43.94	−0.02895	2.255	0.9913
	f_k,ve_	2.084	−10.28	37.39	−0.02818	2.189	0.9873
	f_k,y_	2.411	−9.19	65.77	−0.02225	2.068	0.9879
250 µm	f_k,ip_	2.059	−20.77	67.85	−0.0268	2.126	0.9875
	f_k,ve_	2.142	−11.83	51.35	−0.02598	2.053	0.9788
	f_k,y_	2.384	−15.69	112.9	−0.02297	2.136	0.9953

**Table 5:** Derived characteristic model parameters describing the temperature and strain rate dependence of f_ve_ (MPa) and f_y_ (MPa) of ETFE foils.

Foil Thickness	T (°C)	E (MPa)	E_ve_ (MPa)	K (-)	n (-)	m (-)	Δε_y_ (-)
100 µm	0	1100	90	0.020	7	8	0.05
	23	1000	75	0.035	11	8	0.15
	40	790	60	0.020	15	10	0.08
	50	600	75	0.010	15	10	0.05
250 µm	0	1000	85	0.025	7	11	0.05
	23	1000	75	0.025	11	11	0.05
	40	790	72	0.025	14	9	0.05
	50	510	75	0.015	9	7	0.08

**Table 6:** Comparison of calculated strain using the derived material model versus 5% fractile strain of experimentally determined stress-strain paths of three different producers at specific stresses.

Foil Thickness	T (°C)	$\dot{v}$ (mm/min)	Stress Level (MPa)	ε_calc_ (%)	ε_measured_ (%)	Δε (%)
100 µm	0	10	4.0	0.9	0.9	0.0
			15.0	2.6	2.4	0.2
			20.0	6.5	6.2	0.3
	23	100	4.0	0.4	0.5	-0.1
			15.0	2.6	2.2	0.4
			20.0	9.4	9.1	0.3
	50	500	4.0	0.8	0.8	0.0
			15.0	10.1	9.2	0.9
250 µm	0	10	4.0	0.4	0.7	−0.3
			15.0	1.9	2.2	−0.3
			20.0	5.5	5.2	0.3
	23	100	4.0	0.4	0.4	0.0
			15.0	2.9	2.4	0.5
			20.0	10.6	11.5	−0.9
	50	500	4.0	0.7	0.6	0.1
			15.0	8.3	8.4	−0.1

## Data Availability

The raw/processed data required to reproduce these findings cannot be shared at this time as the data also forms part of an ongoing study. However, it may be made available on request.
